# PREFACE

**DOI:** 10.2174/157015911795017272

**Published:** 2011-03

**Authors:** Syed F Ali, Hyoung-Chun Kim, George Koob, Emmanuel Onaivi, Michael J Kuhar

This volume contains a reviewed selection papers presented at the 2^nd^ International Drug of Abuse Research Society (IDARS) Meeting, a satellite meeting of the International Society for Neurochemistry (ISN) in Association with Korean Drug Abuse Research Society (KDARS). The IDARS/ISN satellite/KDARS meeting entitled "New Research Frontiers and Advances in Drug Addiction” was held on August 14-17, 2009 at the Grand Hyatt Hotel, Seoul, S. Korea. The atmosphere of the meeting especially the surroundings were just magnificent. Over 120 participants from 15 different countries attended the meeting. Owing to a relatively small number of participants, every one enjoyed the scientific exchange in a very relaxing and informal atmosphere. This conference was planned in a way that only one session was held at a time, so no-one missed anything. There was plenty of time allowed for questions and informal discussions.

The major goal of the conference was to understand the cellular and molecular mechanisms of drugs of abuse, such as cocaine, substituted amphetamines (*d*-amphetamine, methamphetamine, and MDMA), alcohol, marijuana, nicotine, opiates, GHB and organic solvents. Separate sessions were devoted to the underlying mechanisms of drug addiction such as genes and drugs of abuse; gene behavior and psychostimulants; substituted amphetamine neurotoxicity; novel neurobiological targets for the treatment of alcoholism, psychostimulants and opiates addiction. Another feature of this conference was that each session included both clinical and basic research scientists working in the same area of research. Several scientists presented clinical and basic studies aimed at understanding the addictive effects of drugs of abuse as well as developing new strategies to treat drug addiction. Although a tremendous body of data has been gathered on the molecular genetics of abused drugs, the molecular mechanisms responsible for addiction and toxicity induced by exposure to these drugs remain to be fully elucidated. The informal atmosphere of the meeting allowed connections and potential collaborations to be established. This occurred especially during the poster session, which was extremely productive. At the end of the conference, there was a panel discussion and open forum, during which a summary of the conference was made and future recommendations voiced. The conference achieved its main goal of bringing together clinical and basic scientists from around the world in a multidisciplinary forum to exchange ideas and data relevant to the expanding field of drug addiction.

We express our gratitude to the organizations and the government agencies that supported this meeting. All papers included in this issue were reviewed by at least one referee, and we would like to thank them all for their valuable time and efforts. We also thank Professor Tom Salt, Editor-in-chief, *Current Neuropharmacology *for publishing these manuscripts. Last, but not least, we want to thank many of our colleagues who helped us in the organizing of this satellite meeting. The 3^rd^ IDARS/ISN-sponsored satellite meeting dealing with the same topic will be held on August 23 – 26, 2011, at Grand Hyatt Hotel in Istanbul, Turkey.

